# 3D-surface scan based validated new measurement technique of femoral joint line reconstruction in total knee arthroplasty

**DOI:** 10.1186/s40634-021-00330-5

**Published:** 2021-02-25

**Authors:** Lars-Rene Tuecking, Max Ettinger, Dennis Nebel, Bastian Welke, Michael Schwarze, Henning Windhagen, Peter Savov

**Affiliations:** 1 Department of Orthopaedic Surgery, Medical School Hannover, Anna-von-Borries-Str. 1-7, 30625 Hannover, Germany; 2 Laboratory for Biomechanics and Biomaterials, Medical School Hannover, Anna-von-Borries-Str. 1-7, 30625 Hannover, Germany

**Keywords:** Total knee arthroplasty, Femoral mechanical angle, Kinematic alignment, Joint line, Radiographic assessment

## Abstract

**Purpose:**

This study aimed to validate a new joint line measurement technique in total knee arthroplasty for separated assessment of the medial and lateral femoral joint line alteration with 3D-surface scan technology. Separate assessment of the medial and lateral joint line alteration may improve TKA alignment assessment regarding to joint line restoration in kinematic alignment and use of robotic-assisted TKA surgery.

**Methods:**

The medial and lateral joint line difference after TKA implantation on an artificial bone model was analyzed and compared with a 3D-scan and full femoral radiographs pre- and postoperatively. Radiographic analysis included the perpendicular distance between the most distal point of the medial and lateral condyle and the reproduced preoperative lateral distal femoral angle (LDFA). For evaluation of validity and reliability, radiographs were captured initially with true anteroposterior view and subsequently with combined flexion and rotation malpositioning. Reliability of the introduced measurement technique in between three observers was tested with intraclass correlation coefficient (ICC).

**Results:**

Radiographic measurement showed a mean difference of 0.9 mm on the medial side and 0.6 mm on the lateral side when compared to the 3D-surface scan measurement. The reliability of measurement accuracy was ≤ 1 mm in x-rays with < 10° flexion error regardless to malrotation in these images. The ICC test showed very good reliability for the medial joint line evaluation and good reliability for lateral joint line evaluation (ICC 0.92, ICC 0.86 respectively).

**Conclusion:**

The new introduced joint line measurement method showed a sufficient reliability, accuracy and precision. It provides separated information about medial and lateral joint line alteration in TKA surgery in absolute values.

**Level of evidence:**

V - Experimental Study

## Introduction

Joint line (JL) restoration is known to be an important factor in total knee arthroplasty (TKA). Several studies have already shown that JL alteration has an impact of clinical and functional outcome [1, 3, 4, 7, 21, 25, 27, 34]. A cadaveric study showed that JL elevation of up to 5 mm could lead to significant increase of mid flexion instability [34]. Additional studies found an alteration of maximum range of motion (ROM) and biomechanics of the patella femoral joint if inaccurate restoration of the JL is apparent [7, 17].

Several studies have already been conducted to define reproducible techniques to quantify absolute JL alteration [15–17, 22, 25, 26, 33, 35]. Most frequently used bony landmarks within these techniques are the adductor tubercle, medial and lateral epicondyle, tip of the fibular head and the tibial tubercle [8, 9, 15, 16, 18, 26, 29, 30]. In recent studies, two principles of JL assessment in native radiographs are used most frequently. The tibial referenced method uses the distance from the tip of the fibula head to the distal femoral joint line [1, 22, 33]. The distance between the level of the adductor tubercle and the distal femoral joint line (ATJL) serves as a femoral referenced method [15, 26]. Both techniques showed good to excellent results for intra- and inter-reliability [1, 15, 16, 30]. The ATJL method provides absolute values of the JL to allow pre- to postoperative JL comparison. Nevertheless, this frequently used method does not allow a differentiation of JL alteration on the medial and lateral side.

Current measurement methods only provide separated measurement methods of the JL height alteration (ATJL height) and alteration of the JL obliquity [11, 12]. The introduced radiographic assessment method in this study combines the assessment of the alteration of the joint line obliquity as well as the absolute joint line height. In addition, the introduced method offers the ability to assess the medial and lateral JL separately. The joint line alteration in the medial and lateral compartment can be expressed in absolute values by this method. This may be particularly beneficial in studies related to kinematic alignment and assessment of knee phenotypes in TKA surgery.

Kinematic alignment aims to restore the native femoral phenotype by reconstruction of the preoperative JL obliquity in addition to the restoration of the JL height itself [19, 24, 27, 28]. The separated determination of postoperative TKA alignment combining JL obliquity and JL height measurement on the medial and lateral side allows a much more detailed postoperative joint line evaluation in knee total arthroplasty research. Complementing this, Hirschmann et al. 2019 introduced the concept of functional knee phenotypes [12] and classified knee phenotypes based on tibial and femoral bony alignment in healthy knee joints [11]. This classification particularly correlates tibial and femoral bony alignment. In these studies, a high variability of knee phenotypes was found in healthy knee joints. Thus, this lead to the conclusion that the usual classification of knee joints based only on the overall limb alignment into three groups (varus, valgus, neutral) does not correctly reflect the natural variability of knee joints. Therefore, it is necessary to apply patient-specific alignment philosophies to reconstruct the natural alignment and natural knee phenotype. However, this requires new measurement methods that allow detailed postoperative assessment of the alignment and the femoral phenotype. The separate assessment of the medial and lateral joint line allows a much more detailed assessment of the postoperative alignment and might be used to combine the assessment of knee phenotypes in line with JL alteration. Additionally, robotic-assisted surgery is used more frequently in knee arthroplasty. Several studies could already show an increased accuracy in TKA alignment with an increased control of femoral bone cuts of the medial and lateral condyles in TKA procedures [2, 31]. Hence, a JL assessment method with separated measurement of the medial and lateral JL might also be beneficial in this context.

This study introduced a new JL measurement technique on long leg radiographs with separated absolute values for medial and lateral joint line alteration. It leads to an accurate evaluation of the success of modern alignment philosophies and precision of robotic assisted TKA.

This study is the first to introduce a JL measurement technique which combines the assessment of JL obliquity and JL height, and offers a separated medial and lateral JL assessment. This technique offers the possibility of a very precise analysis of the postoperative femoral alignment, which has a special value in studies of kinematic alignment or, in the future, in accuracy studies of robotic assisted TKA.

Thus, the purpose of this study is twofold. First of all, this study was performed to validate the measurement technique by the use of a modern 3D-scan technology. Further, the resistance of the new JL measurement technique to typical rotation and extension errors in long leg radiographs was investigated. It was hypothesized that the newly introduced JL measurement is accurate about one mm in the assessment of medial and lateral JL and is resistant to slight misplacement within radiographic imaging.

## Material and methods

A femoral artificial bone (SKU: 1103–1, Sawbones Europe, Sweden) was used as a testing model in this radiographic validation study.

### Implant positioning

A cruciate retaining femoral component (Size 5, Stryker Triathlon; Stryker, USA) was positioned with kinematic alignment technique using the standard instruments [14]. The medial and lateral bone cuts were symmetrical to reproduce the native anatomy of the femoral model with the implanted femoral component.

### Radiographic assessment

**Fig. 1:**
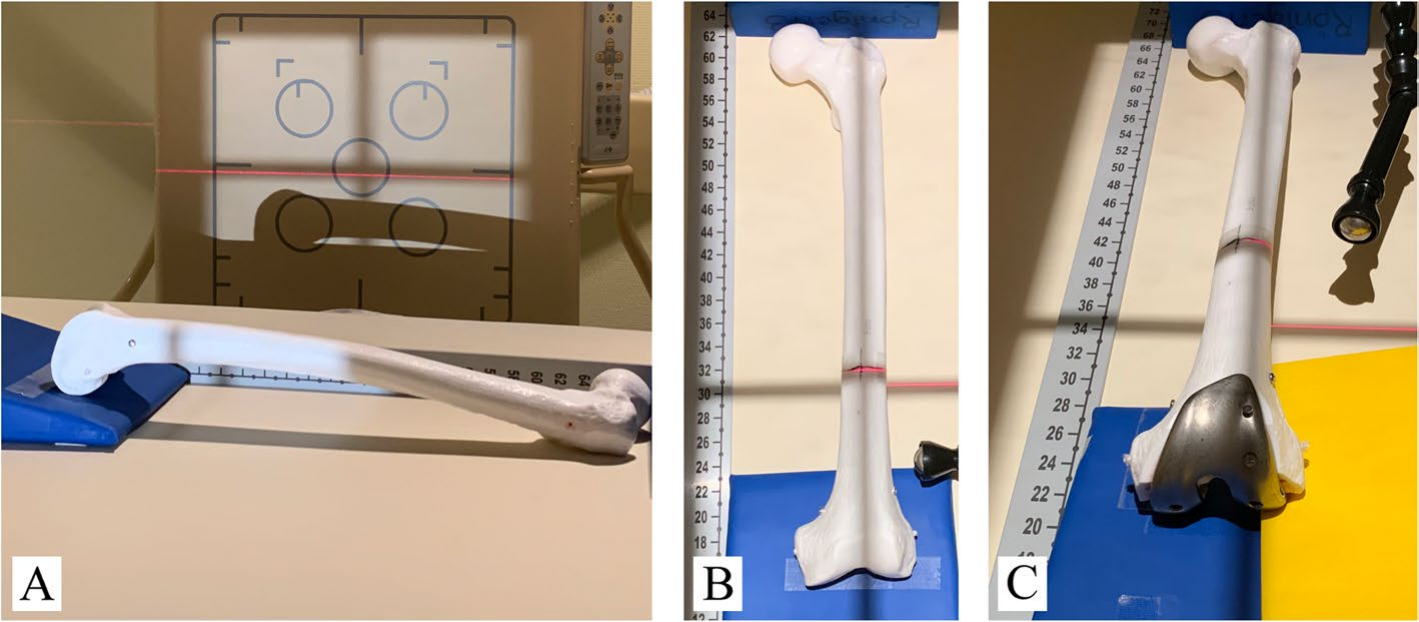
Experimental setup of radiographic imaging with validation and setup of differenct error conditions. Flexion error validation in true lateral view (**a**). A.p. view preoperative with 5° flexion error (**b**). A.p. view postoperative with internal rotation error (**c**). Laser is used to verify bone position on X-ray table. Calibration bar and calibration ball for internal validation of distance measurement in PACS software

**Fig. 2:**
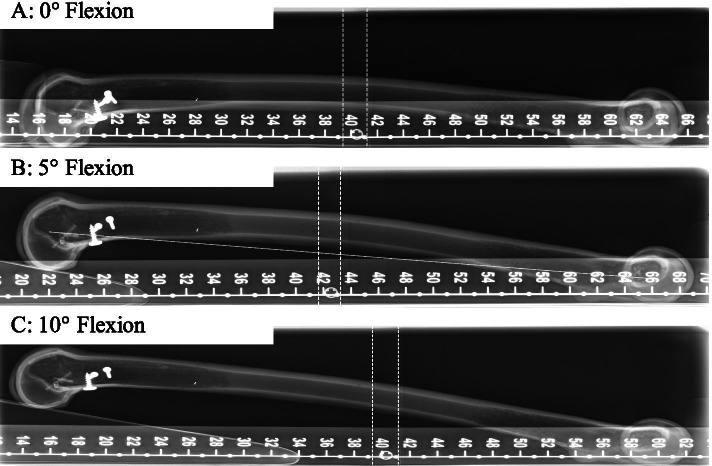
Experimental setup of radiographic imaging for flexion error. Different flexion error setups with 0° flexion error (**a**), 5° flexion error (**b**) and 10° flexion error (**c**). Scale bar used for angle measurement and calibration

Radiographic assessment was conducted pre- and postoperatively with the same test protocol and set-up (see Fig. 1). Simulation of X-ray malpositioning included 5° and 10° flexion error in a.p. views and additional rotational error with 5° internal and 5° external rotation were added to each neutral a.p. view (see Table 2). The set-up of the flexion error was validated by true lateral views (Fig. 2) and consecutive measurement within the PACS software. A goniometer was used to reproduce and verify internal and external rotation of 5°. The use of radio translucent positioning devices fixed to the X-ray table on defined positions as well as the use of optical positioning aids ensured repeatability of the setup and comparability of pre- and postoperative X-rays. Although the used X-ray machine (DigitalDiagnost Dual Detector, Philips, Netherlands) already provides calibrated images, a calibration ball and calibration scales were added to every image to confirm the distance calibration.

### 3D-scan assessment

**Fig. 3:**
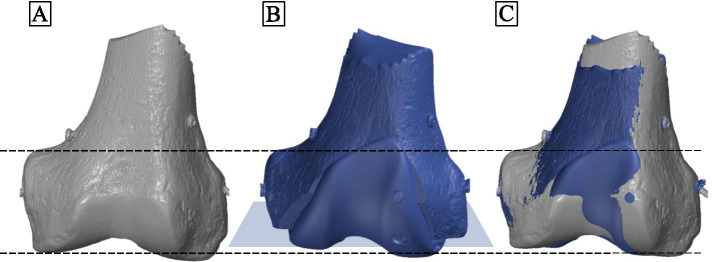
3D-scan based evaluation of the distal femur pre- and postoperative. Preoperative (**a**) and postoperative (**b**) 3D-scan of the distal femur and overlay of both scans (**c**). Blue square (**b**) illustrates the plane of the distal femoral cuts

3D-surface scans were taken pre- and postoperatively. Initially, the testing model was sprayed with matting spray (3D Scanning Mattierungsspray FabConstruct L500, Fabistron GmbH, Zerbst, Germany) in order to increase the contrast and to avoid scan artefacts. The artificial bone was then placed on a turntable (HP 3D Automatic Turntable Pro, HP Deutschland GmbH, Böblingen, Germany) of the associated 3D-scanner system (HP 3D Structured Light Scanner Pro S3, HP Deutschland GmbH, Böblingen, Germany). After the first 360° scan (increments of 30 degree) the model was repositioned for another scan to detect the whole surface, including the distal joint surface of the femur. The single scan frames were recorded by the scan software (HP 3D Scan Pro 5.6.0.2037 Rev. I (17.01.2019), HP Deutschland GmbH, Böblingen, Germany), which was also used to align the frames and merge them into a 3D object. This resulted in triangulated surface models with around 3.7 million faces and an average edge length of 0.12 mm (Fig. 3). For the 3D analysis the pre- and postoperatively 3D objects were imported into GOM Inspect (GOM Inspect 2019 (2019 Hotfix 3, Rev. 121,775, Build 2019–10-10), GOM GmbH, Braunschweig, Germany). Initially, the two objects were superimposed in GOM by 3-point alignment of reference points and subsequent local best-fit of the unaffected bone surface. Afterwards, a plane perpendicular to the distal femoral bone cuts from the postoperative 3D-object was created. The created plane was used to create a 2D silhouette of the objects. The most distal points on the medial and lateral side of the femur were then selected, as well as the medial adductor tubercle. All measurements were repeated three times by one engineer. The mean value was used for further statistical analysis.

### Radiological analysis

**Fig. 4:**
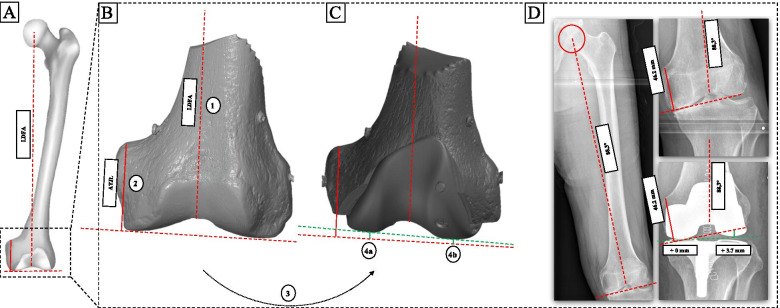
Schematic illustration of radiographic measurement of medial and lateral joint line difference. Schematic illustration of a left femur with measurement of lateral distal femoral angle (LDFA) and adductor tubercle joint line distance (ATJL) (**a**). Preoperative (**b**) and postoperative distal femur (**c**). Preoperative LDFA (dashed) and ATJL (continuous) illustrated by red lines in (**b** and **c**). Green line shows postoperative joint line and perpendicular distance from most distal medial and lateral points to preoperative joint line. 1.) Measurement of preoperative LDFA in °, 2.) measurement of preoperative ATJL in mm as perpendicular distance from the adductor tubercle and the preoperative joint line (≜ LDFA), 3.) reconstruction of the preoperative LDFA and ATJL in the postoperative radiograph and 4.) measurement of the medial (**a**, mJLD) and lateral (**b**, lJLD) distance between the most distal point and the preoperative joint line. Example measurement (**d**) of the medial and lateral joint line alteration including example values of the ATJL, LDFA, mJLD and lJLD

For radiological analysis Carestream PACS software V.11 (Carestream, Philips, Netherlands) was used. The preoperative assessment included measurement of the lateral distal femur angle (LDFA) and the perpendicular distance between the medial adductor tubercle and the distal femoral joint line (ATJL, preoperative joint line height) as described before [32]. The preoperative LDFA and ATJL of the reference radiograph (true a.p., neutral rotation) were then reproduced in each postoperative radiograph. Within the postoperative radiograph the perpendicular distance between the most distal point of the medial and lateral condyle and the reproduced preoperative LDFA (preoperative joint line) served as measurement of the medial joint line difference (mJLD) and lateral joint line difference (lJLD) as shown in Fig. 4. Three independent observers (orthopedic surgeons) carried out the radiological analysis.

### Statistical analysis

Statistical analysis was performed using the software SPSS v25 (IBM, Armonk, NY, USA) and Microsoft Excel (Microsoft, Washington, USA). Descriptive results are given as mean value, standard deviation, median and minimum and maximum values (Table 1). Accuracy of radiographs with rotation and flexion error were calculated as difference to the reference radiograph (neutral, true a.p.; Table 2). Inter-observer reliability was calculated with the intraclass correlation coefficient (ICC). An ICC value > 0.8 was defined as good reliability, values > 0.9 as very good reliability.

**Table 1:** Comparison of radiographic and 3D-surface scan measurements: JL difference pre- to postoperative

	**Mean**	**SD**	**Median**	**Max**	**Min**
**Radiographic analysis**^**a**^					
Medial JL difference (mm)	**-2.2**	± 0.1	-2.2	-2.0	-2.3
Lateral JL difference (mm)	**-0.4**	± 0.3	-0.6	0.5	-0.6
**3D Structured Light Scanner analysis**					
Medial JL difference (mm)	**-1.3**	± 0.1	-1.2	-1.2	-1.4
Lateral JL difference (mm)	**0.2**	± 0.1	0.2	0.3	0.1
**Difference in between techniques**					
**Medial JL difference (mm)**	**-0.9**		**-1.0**		
**Lateral JL difference (mm)**	**-0.6**		**-0.8**		

*LDFA* Lateral distal femoral angle, *JL* Joint line, *SD* standard deviation, *Max* Maximum, *Min* Minimum, ^a^0° Flexion without rotation error (reference measurement), negative sign expresses ‘proximalisation’

**Table 2:** Radiographic measurements of LDFA and ATJL with flexion and rotation error

Flexion and rotation error		Preoperative LDFA (°)	Preop. LDFA diff. to reference (°)	Postoperative LDFA (°)	Postop. LDFA diff. to reference (°)	Preoperative ATJL (mm)	ATJL diff. to reference (mm)	Postoperative ATJL (mm)	Postoperative ATJL diff. to reference (mm)
**0° flexion**	**+0° rotation**	**91.6 (**± **0.2)**		**93.7 (**± **0.3)**		**49.3 (**± **0.1)**		**46.9 (**± **0.4)**	
0° flexion	+5° internal rotation	92.4 (± 0.3)	0.9 (± 0.3)	94.9 (± 0.3)	1.2 (± 0.2)	48.9 (± 0.3)	-0.4 (± 0.3)	46.9 (± 0.2)	0.0 (± 0.3)
0° flexion	+5° external rotation	90.7 (± 0.3)	-0.9 (± 0.3)	92.8 (± 0.3)	-0.9 (± 0.1)	49.9 (± 0.9)	0.2 (± 0.2)	47.4 (± 0.1)	0.5 (± 0.4)
**5° flexion**	**+0° rotation**	**91.8 (**± **0.2)**	**0.3 (**± **0.2)**	**93.7 (**± **0.1)**	**0.0 (± 0.2)**	**49.4 (**± **0.3)**	**0.2 (**± **0.4)**	**47.8 (**± **0.1)**	**0.9 (± 0.3)**
5° flexion	+5° internal rotation	91.9 (± 0.1)	0.3 (± 0.1)	93.9 (± 0.1)	0.2 (± 0.1)	49.3 (± 0.6)	0.0 (± 0.6)	48.3 (± 0.2)	1.4 (± 0.2)
5° flexion	+5° external rotation	91.7 (± 0.7)	0.1 (± 0.5)	93.2 (± 0.2)	-0.5 (± 0.1)	49.8 (± 0.6)	0.5 (± 0.6)	48.1 (± 0.1)	1.2 (± 0.4)
**10° flexion**	**+0° rotation**	**92.3 (**± **0.2)**	**0.7 (**± **0.1)**	**93.8 (**± **0.3)**	**0.1 (± 0.1)**	**49.5 (**± **0.5)**	**0.2 (**± **0.6)**	**49.1 (**± **0.2)**	**2.2 (± 0.2)**
10° flexion	+5° internal rotation	92.1 (± 0.1)	0.5 (± 0.3)	93.5 (± 0.3)	-0.2 (± 0.3)	49.6 (± 0.6)	0.3 (± 0.7)	49.1 (± 0.3)	2.2 (± 0.2)
10° flexion	+5° external rotation	92.3 (± 0.4)	0.7 (± 0.2)	93.9 (± 0.2)	0.2 (± 0.4)	49.9 (± 0.7)	0.6 (± 0.7)	49.2 (± 0.3)	2.3 (± 0.1)

*LDFA* Lateral distal femoral angle, *JL* Joint line, *med*. medial, *lat*. lateral, *diff*. difference, values given in mean (± standard deviation)

## Results

The radiographic measurement technique showed a total mean difference of 0.9 mm on the medial side and 0.60 mm on the lateral side compared to the 3D-surface scan (Table 1). A further proximalisation of the joint line was apparent at both sides when compared to the 3D-surface scan technique.

**Fig. 5:**
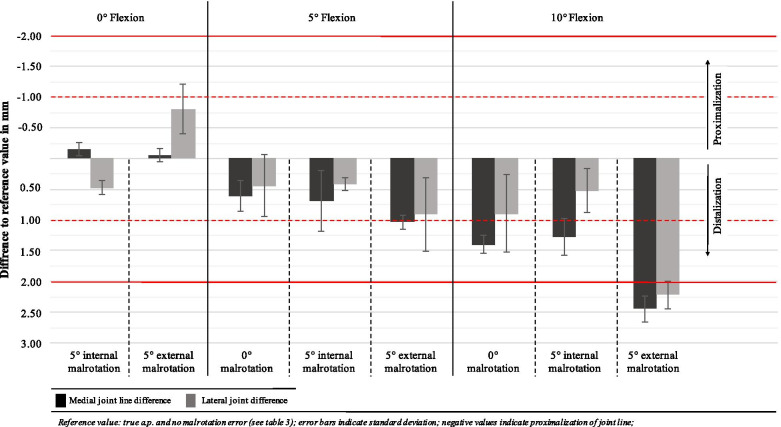
Differences to reference values of medial and lateral joint line difference

Radiographic malpositioning influenced the medial and lateral JL evaluation with a range of difference of -0.4 mm to 2.5 mm (medial side) from the reference radiograph, and -0.8 mm to 2.5 mm on the lateral side (Table 3, Fig. 5). The highest deviation from the reference radiograph was found for 10° flexion error with 5° external rotation (medial: 2.5 mm and lateral: 2.5 mm). The difference from the reference radiograph did not exceed ≤ 1 mm on the medial or lateral side if flexion error was kept ≤ 10° (Fig. 5).

**Table 3:** Radiographic measurements of medial and lateral JL difference with flexion and rotation error

Flexion and rotation error	Medial JL difference (mm)	Lateral JL difference (mm)
**0° flexion (reference)**	**-2.2 (**± **0.1)**	**-0.4 (± 0.3)**
+ 5° internal rotation	-2.5 (± 0.2)	0.1 (± 0.2)
+ 5° external rotation	-2.2 (± 0.1)	-1.2 (± 0.4)
**5° flexion**	**-1.5 (**± **0.3)**	**0.0 (± 0.5)**
+ 5° internal rotation	-1.5 (± 0.5)	0.4 (± 0.6)
+ 5° external rotation	-1.1 (± 0.1)	0.6 (± 0.6)
**10° flexion**	**-0.8 (**± **0.2)**	**0.6 (± 0.7)**
+ 5° internal rotation	-0.8 (± 0.3)	0.3 (± 0.7)
+ 5° external rotation	0.3 (± 0.2)	2.1 (± 0.4)

*LDFA* Lateral distal femoral angle, *JL* Joint line, *med*. medial, *lat*. lateral, *diff*. difference, values given in mean (± standard deviation)

Preoperative LDFA (91.6° ± 0.2°) changed by 2.1° to the postoperative LDFA (93.7° ± 0.3°), see Table 2. When assessing the influence of the malpositioning, the LDFA difference to the reference were mostly kept < 1° with one exception (postoperative LDFA with 0° flexion and 5° internal malrotation), which at the same time showed the largest difference to the reference (1.2°).

Within the ATJL assessment, the difference to the neutral radiograph ranged from -0.4 mm to 0.6 mm (preoperative) and from 0.01 mm to 2.3 mm (postoperative). The maximum differences to the neutral positioning were found in the group of 10° flexion and 5° external malpositioning with 0.6 mm (preoperative) and 2.3 mm (preoperative).

The reliability of the medial joint line difference measurement was proven very good with an ICC of 0.92 and of the lateral joint line a good reliability with an ICC of 0.86.

## Discussion

The most important finding of this study is that the newly defined JL assessment method shows a high accuracy with a deviation of ≤ 1 mm from 3D-surface scan measurement. This error is acceptable within the limits of clinical relevance. This JL assessment method provides the possibility of differentiated measurement of medial and lateral JL alteration. Additionally, the validity of this method was proven by resilience against typical malalignments in certain limits during radiograph acquisition. However, an extension deficit of 10° with a combined external malrotation lead to a high deviation of > 2 mm from the reference radiograph. Nevertheless, an external malrotation with an extension deficit of 10° is obvious to the examiner and should lead to consider repetition of the radiographs. Therefore, this limitation of the assessment method is acceptable in clinical and scientific practice.

The aim of kinematic alignment is to exactly restore the natural anatomy and the extension-flexion axis of the femur which includes the restoration of the joint line and its obliquity [6, 19, 24]. No JL assessment method has yet been described and validated, which differentiate between medial and lateral JL alteration in absolute values. With strict adherence to the kinematic alignment, a symmetrical postoperative medial and lateral JL change should be achieved. In contrast, the treatment of a valgus osteoarthrosis with a correction of the overall limb alignment should show a greater distalization of the lateral JL compared to the medial JL. However, an evaluation of the intended targets cannot be made with the current available assessment methods.

Robotic-assisted knee arthroplasty has proven increased alignment accuracy and decreased bone loss [2, 23, 31]. Sires et al. could show an accuracy of bone cuts < 1 mm in 94.3% of the cases performed with an imaged-based robotic system [31]. This suggests that amongst other parameters the control of the medial and lateral JL could be improved by robotic-assisted surgery. Therefore, the analysis of medial and lateral JL alteration might be beneficial for accuracy analysis in robotic-assisted surgery.

Furthermore, there is still a lack of a standardized measurement procedure in TKA. In a systematic review by van Lieshout et al. recent studies with regards to joint line elevation after TKA were analyzed [33]. The majority of the authors rely on three different bony landmarks as a reference point to calculate the joint line height: the fibular head, the tibial tubercle and the adductor tubercle [33]. Several authors already showed that the fibular head is not a reliable bony landmark for JL assessment [9, 15, 30]. In this context, the adductor tubercle seems to show the highest reliability [15, 16, 26]. Therefore, the study group modified an existing assessment method, which is based on femoral landmarks and uses the adductor tubercle as the reference point [1, 13, 20, 32]. Based on the JL measurement technique in unicompartimental knee arthroplasty (UKA), a second reference value was added: the preoperative joint line obliquity [10]. These two factors leads to a differentiate evaluation between the two compartments of the femur.

The accuracy of the proposed method is promising with ≤ 1 mm deviation to a 3D scan. These error values are within clinical acceptance and get close to the threshold value (1 mm) found in robotic-assisted TKA studies [31].

The ICC shows a good and very good correlation for the radiographic measurement between all examiners. These results are comparable to those, which are published for standard limb alignment on long leg x-rays (ICC = 0.7 to 0.8) [5].

A major problem in examination of radiographs is the varying quality of the images [5]. Especially, the assessment of the mechanical axis in early postoperative long leg radiographs is dependent on the limb loading [36]. In order to test the influence of malposition of patients knee on the described JL assessment method, different common flexion and rotation errors were simulated (Fig. 1 and 2). The results suggest that the limit for the evaluation of x-ray images with this technique is 10° flexion in combination with external rotation or more than 10° of flexion without rotational malpositioning. Furthermore, the difference to the reference radiograph was ≤ 1 mm in x-rays with < 10° flexion error regardless to malrotation in these images (Fig. 5). Thus, the new measuring method described in this study can be used in everyday clinical practice without greater concerns since an extension deficit of more than 10° can obviously be detected in the x-ray image.

This current study has several limitations. All of our findings are based on one saw bone femur model. The adductor tubercle of the saw bone model is less prominent as found in natural bones. In our opinion, the repeated identification of the adductor tubercle in the given x-rays is the most crucial step in this measurement method. This might even have influenced the values of the ICC negatively. In this study, only radiographs of the femoral sawbone taken in the supine position were used, which, of course, cannot accurately represent the appearance of the native femur in weight-bearing images. Nevertheless, it was tried to position the sawbone as natural as possible by comparison with whole-leg radiographs taken in the supine position with the same X-ray unit. Further, the focus of this study was to evaluate and validate the measurement method in comparison to 3D scan technology. Clinical data on a study cohort is missing, due to the focus of this study. Therefore, no conclusion on the clinical effect on different values of medial or lateral joint line alteration is possible.

## Conclusion

The new introduced joint line measurement method showed a sufficient reliability, accuracy and precision. It provides separated information about medial and lateral joint line alteration in TKA surgery in absolute values.

## Abbreviations

ATJL: Adductor tubercle to joint line distance
a.p.: Anteroposterior
diff.: Difference
Fig.: Figure
ICC: Intraclass correlation coefficient
JL: Joint line
KA: Kinematic alignment
lat.: Lateral
LDFA: Lateral distal femoral angle
lJLD: Lateral joint line difference
med.: Medial
mJLD: Medial joint line difference
ROM: Range of motion
TKA: Total knee arthroplasty
